# Primary care-based, targeted screening programme to promote sustained weight management

**DOI:** 10.3109/02813432.2014.886493

**Published:** 2014-03

**Authors:** Päivi E. Korhonen, Salme Järvenpää, Hannu Kautiainen

**Affiliations:** ^1^Institute of Clinical Medicine, Family Medicine, University of Turku, Turku, Finland; ^2^Satakunta Hospital District, Pori, Finland; ^3^Central Satakunta Health Federation of Municipalities, Harjavalta, Finland; ^4^Medcare Foundation, Äänekoski, Finland; ^5^Unit of Primary Health Care, Helsinki University Central Hospital, Helsinki, Finland; ^6^Department of General Practice, University of Helsinki, Helsinki, Finland

**Keywords:** Finland, general practice, lifestyle counselling, obesity, overweight, primary care, screening, weight management

## Abstract

*Objective*. To identify overweight and obese subjects at increased cardiovascular risk in the community, and provide them with lifestyle counselling that is possible to implement in real life. *Design*. Longitudinal cohort study. *Setting*. The communities of Harjavalta and Kokemäki in south-western Finland. *Subjects*. A tape for measurement of waist and a risk factor questionnaire was mailed to home-dwelling inhabitants aged 45–70 years (n = 6013). Of the 4421 respondents, 2752 with at least one cardiovascular risk factor were examined by a public health nurse. For the subjects with high cardiovascular risk (n = 1950), an appointment with a physician was scheduled. The main goal of lifestyle counselling for the 1608 high-risk subjects with BMI ≥ 25 kg/m^2^ was weight reduction of at least 5%. Among these, 906 had completed self-administrated questionnaires at baseline and form the present study population. *Main outcome measure*. Success in weight management. *Results*. At the three-year follow-up visit, 18% of subjects had lost ≥ 5% of their initial weight and 70% had stabilized their weight, while 12% had gained weight ≥ 5%. Newly diagnosed glucose disorder (OR 1.37 [95% CI 1.02–1.84]) predicted success in weight management, whereas depressive symptoms (OR 0.61 [95% CI 0.42–0.90]), excess alcohol use (OR 0.63 [95% CI 0.44–0.90]), and number of drugs used (OR 0.91 [95% CI 0.83–0.99]) at baseline predicted poor outcome. *Conclusions*. A primary care screening programme to identify overweight or obese individuals can promote sustained weight management. Psychological factors, especially depressive symptoms, are a critical component to consider before attempts to change the lifestyle of an individual.

It is unknown whether a primary care-based obesity screening programme can promote long-term success in weight management.

- Using a targeted screening strategy together with low-intensity counselling, sustained weight management can be achieved in a primary care setting.
- Newly diagnosed glucose disorder seems to be an important motivating factor for weight loss.
- Of the participants with a weight gain of 5% or more during the follow-up, one in three had depressive symptoms at baseline.

## Introduction

There is an urgent need for new strategies to prevent the global epidemic of obesity and diabetes and subsequent rise in cardiovascular diseases. Key issues in the prevention of these conditions are to find the persons at risk and assess their willingness to maintain lifestyle changes in the long term.

A recent systematic review identified no trials of primary care screening programmes for obesity or overweight in adults reporting improved physiologic outcomes or those which resulted in weight loss [1]. Most clinical studies have used body mass index (BMI) to assess excess adiposity, although BMI has poor sensitivity to detect adiposity versus lean muscle mass and body fat distribution.

A targeted, two-stage screening strategy, the Harmonica project, was conducted in south-western Finland in 2005–2007 to identify 45- to 70-year-old persons at risk of type 2 diabetes and cardiovascular diseases in the general population. Waist circumference home measurement together with a risk factor questionnaire was used as a primary screening tool. The present study expands on this screening phase by assessing the success of brief lifestyle counselling delivered at baseline in a three-year follow-up of the overweight and obese subjects identified at screening.

## Material and methods

### Participants and the design of the Harmonica project

The subjects of the study were participants in a population survey, the Harmonica Project, which was carried out in the rural towns of Harjavalta and Kokemäki in south-western Finland from autumn 2005 to autumn 2007. A cardiovascular risk factor survey, tape for the measurement of waist circumference, and type 2 diabetes risk assessment form (Finnish Diabetes Risk Score, FINDRISC, available from http://www.diabetes.fi/english), were mailed to all home-dwelling inhabitants aged 45–70 years (n = 6013) [2]. Of the 4421 (74%) respondents, those having at least one cardiovascular risk factor (n = 3072) were invited for an enrolment examination performed by a public health nurse. Risk factors taken into account were waist circumference ≥ 80 cm in women and ≥ 94 cm in men, hypertension, history of gestational diabetes or hypertension, family history of premature cardiovascular disease, and ≥ 15 points in the FINDRISC (≥ 12 points in Harjavalta). Participation and all the tests included were free of charge for the subjects. Patients with known cardiovascular disease or previously diagnosed diabetes were not invited because they already had systematic follow-up in the health centres.

The public health nurses examined 2752 at-risk subjects, explained the test results, and gave verbal and written lifestyle recommendations to all subjects personally. For the subjects with BMI ≥ 25 kg/m^2^ (n = 2147), the main goal of lifestyle counselling was weight reduction of at least 5% by reducing saturated fat in the diet and increasing physical activity to at least 30 minutes per day or four hours per week.

If the test results revealed elevated cardiovascular risk defined as hypertension, glucose disorder, metabolic syndrome diagnosed by the International Diabetes Federation criteria [3], BMI ≥ 30 kg/m^2^, or the 10-year risk of cardiovascular death yielded 5% or more based on the SCORE system [4], an appointment with a physician was scheduled after 2–4 months. The physician repeated lifestyle counselling and assessed the need for preventive medication. There were 1950 subjects with elevated cardiovascular risk, of whom 1608 had a BMI ≥ 25 kg/m^2^.

### Post-intervention follow-up at year three

The subjects with elevated cardiovascular risk were invited to a three-year follow-up visit with the public health nurse. Of the 1287 high-risk subjects who attended, 1049 had filled in the questionnaires completely, and 906 of them had BMI ≥ 25 kg/m^2^ at baseline. These overweight or obese subjects were categorized according to their success in weight management: (i) weight loss ≥ 5%; (ii) stable weight (less than 5% change in weight); (iii) weight gain ≥ 5%. Weight loss of 5% is considered clinically meaningful [5].

### Measurements

Height and weight were measured by a public health nurse with the subjects in standing position without shoes and outer garments. Height was recorded to the nearest 0.5 cm and weight to the nearest 0.1 kg. Digital scales (Seca^®^ 861, Germany) were used, and their calibration was monitored regularly. BMI was calculated as weight (kg) divided by the height squared (m^2^). Waist circumference was measured by a trained nurse at the level midway between the lower rib margin and the iliac crest. The subjects were asked to breathe out gently during the measurement. The tape was held firmly in a horizontal position.

At baseline, plasma glucose levels and lipid profiles were determined in blood samples that were obtained after at least 12 hours of fasting. An oral glucose tolerance test was performed by measuring fasting plasma glucose and two-hour plasma glucose from capillary blood after ingestion of a glucose load of 75 g anhydrous glucose dissolved in water. Glucose disorders were classified according to the World Health Organization 2006 criteria [6]. On the basis of two-hour post-load plasma glucose, individuals were classified into categories of newly diagnosed diabetes, impaired glucose tolerance, and normal glucose tolerance if their two-hour plasma glucose concentrations were ≥ 12.2, 8.9 to12.1, and < 8.9 mmol/l, respectively. Impaired fasting glucose was diagnosed if the fasting plasma glucose was ≥ 6.1 mmol/l and the two-hour plasma glucose was < 8.9 mmol/l.

Blood pressure was measured by a trained nurse with a calibrated mercury sphygmomanometer with subjects in a sitting posture, after resting for at least five minutes. In each subject the mean of the two readings taken at intervals of at least two minutes was used. Hypertension was defined as the use of antihypertensive medication, or as the mean of home blood pressure monitoring ≥ 135 mmHg for systolic or ≥ 85 mmHg for diastolic blood pressure [7].

### Questionnaires

Subjects completed self-administrated questionnaires at the baseline visit: sociodemographic factors, occupational status, physical-activity level, smoking status, Alcohol Use Disorders Identification Test (AUDIT) [8], and Beck's Depression Inventory (BDI) [9]. An AUDIT score of 8 or more indicates harmful alcohol use, as well as possible alcohol dependence [8]. Depressive symptoms were regarded as present if the BDI score was ≥ 10 [10].

Leisure-time physical activity (LTPA) was classified as follows: (i) high: LTPA ≥ 30 minutes at a time at least six times a week; (ii) moderate: LTPA ≥ 30 minutes at a time from four to five times a week; (iii) low: LTPA ≥ 30 minutes at a time three times a week or less.

### Informed consent

The study protocol and consent forms were reviewed and approved by the ethics committee of Satakunta hospital district. All participants provided written informed consent for the project and subsequent medical research.

### Statistical analysis

The data are presented as means with standard deviations (SD) or as medians with interquartile range (IOR) or as counts with percentages. The 95% confidence intervals are given for the most important outcomes.

Statistical comparison in characteristics between followed-up and not followed-up subjects was made by chi-squared test, *t*-test or Mann–Whitney test. Statistical comparisons in baseline characteristics between study groups were made by analysis of variance, Kruskal–Wallis test, or chi-squared test.

To determine characteristics associated with weight-loss success, univariate and multivariate forward stepwise ordered logistic regression analysis was applied. Age-adjusted relative weight changes were estimated and tested between groups by using a random-effects regression model to longitudinal data with an appropriate contrast.

## Results

We examined 2147 screen-detected overweight or obese cardiovascular risk subjects, of whom 2100 had adequate data available on questionnaires. Three-year follow-up data consisted of 906/2147 (42%) individuals. Table I displays the characteristics of the subjects who attended follow-up examination compared with those who did not. Followed-up subjects were practically of the same age, and had only slightly higher BMI, blood pressure, and LDL cholesterol than those who were not followed up.

**Table I.** Baseline characteristics of the screened overweight and obese subjects according to follow-up category.

	Followed-up (n = 906)	Not followed-up (n = 1194)	p-value
Age, years, mean (SD)	59 (7)	58 (7)	< 0.001
Female, n (%)	476 (53)	634 (53)	0.80
Married or cohabiting, n (%)	692 (79)	907 (78)	0.70
Education years	10.8 (2.3)	10.9 (2.3)	0.23
Employment, n (%)			0.060
Employed	447 (49)	647 (54)	
Retired	403 (45)	470 (39)	
Unemployed	56 (6)	77 (7)	
Body mass index, kg/m^2^, mean (SD)	30.6 (4.3)	30.2 (4.7)	0.016
Blood pressure, mmHg, mean (SD)			
Systolic	143 (18)	140 (19)	0.0024
Diastolic	86 (10)	85 (11)	0.0066
Total cholesterol, mmol/l, mean (SD)	5.43 (0.99)	5.37 (1.04)	0.18
LDL cholesterol, mmol/l, mean (SD)	3.31 (0.88)	3.23 (0.86)	0.056
HDL cholesterol, mmol/l, mean (SD)	1.47 (0.39)	1.51 (0.45)	0.037
Triglycerides, mmol/l, mean (SD)	1.50 (0.71)	1.47 (1.18)	0.56
Fasting glucose, mmol/l, mean (SD)	5.59 (0.83)	5.78 (1.46)	< 0.001
2-hour glucose, mmol/l, mean (SD)	7.62 (2.19)	7.52 (2.58)	0.33
LTPA, n (%)			0.41
Low	353 (40)	430 (38)	
Moderate	263 (30)	366 (32)	
High	258 (30)	350 (30)	
BDI score, mean (SD)	5.8 (5.2)	5.9 (6.0)	0.67

Notes: LDL = low-density lipoprotein; HDL = high-density lipoprotein; LTPA = leisure-time physical activity; BDI = Beck's Depression Inventory.

Table II shows the baseline characteristics of the subjects according to their success in weight management at the three-year follow-up. Those 163/906 (18%) who had lost ≥ 5% of their initial weight had more often newly detected diabetes, higher blood pressure values, and lower AUDIT scores at baseline than the other study groups. Those 106/906 (12%) who had gained ≥ 5% weight at year three were less frequently married or cohabiting, had depressive symptoms and used antidepressant medication more often than those who managed to lose or stabilize their weight.

**Table II.** Baseline characteristics of the subjects according to their success in weight management.

	Weight loss ≥ 5% (n = 163)	Stable weight (n = 637)	Weight gain ≥ 5% (n = 106)	p-value
Female, n (%)	90 (55)	320 (50)	66 (62)	0.054
Age, years, mean (SD)	59 (7)	59 (7)	58 (6)	0.29
Waist, cm, mean (SD): Female Male	99 (10) 105 (10)	97 (11) 104 (10)	98 (12) 104 (10)	0.31 0.62
Married or cohabiting	120 (76)	500 (81)	72 (69)	0.012
Education years, mean (SD)	10.7 (2.3)	10.8 (2.3)	10.7 (2.5)	0.72
Employment, n (%):				0.48
Employed	78 (48)	317 (50)	52 (49)	
Retired	79 (48)	279 (44)	45 (42)	
Unemployed	6 (4)	41 (6)	9 (8)	
Body mass index, kg/m^2^, mean (SD)	31.1 (4.5)	30.5 (4.3)	30.6 (4.6)	0.37
Blood pressure, mmHg, mean (SD): Systolic Diastolic	144 (18) 86 (10)	143 (18) 86 (10)	137 (16) 84 (9)	0.0012 0.042
Total cholesterol, mmol/l, mean (SD)	5.46 (0.97)	5.45 (1.00)	5.30 (0.99)	0.31
LDL cholesterol, mmol/l, mean (SD)	3.32 (0.80)	3.32 (0.90)	3.17 (0.89)	0.23
HDL cholesterol, mmol/l, mean(SD)	1.48 (0.44)	1.47 (0.38)	1.49 (0.37)	0.87
Triglycerides, mmol/l, mean (SD)	1.52 (0.72)	1.50 (0.69)	1.45 (0.75)	0.76
Glucose homeostasis, n (%):				0.011
Normal	58 (36)	255 (40)	51 (48)	
IFG	58 (36)	234 (37)	36 (34)	
IGT	32 (20)	128 (20)	13 (12)	
T2D	15 (9)	20 (3)	6 (6)	
Current smoker, n (%)	25 (15)	85 (14)	23 (22)	0.094
AUDIT, mean (SD)	3.80 (4.33)	5.01 (4.98)	5.27 (5.81)	0.015
LTPA, n (%):				0.99
Low	64 (41)	246 (40)	43 (41)	
Moderate	47 (30)	187 (31)	29 (28)	
High	46 (29)	180 (29)	32 (31)	
BDI score, mean (SD)	5.3 (5.1)	5.6 (5.1)	7.6 (5.8)	0.0025
BDI score ≥ 10	24 (15)	101 (16)	34 (33)	< 0.001
Current medication:				
Beta-blockers	30 (18)	153 (24)	24 (22)	0.31
Diuretics	15 (19)	72 (11)	11 (10)	0.73
Statins	17 (10)	95 (15)	17 (16)	0.29
Antidepressants	7 (4)	22 (3)	17 (16)	< 0.001
NSAIDs in regular use	5 (3)	13 (2)	4 (4)	0.47
Number of drugs used, median, (IQR)	1 (0–2)	1 (0–2)	2 (1–3)	0.0024

Notes: LDL = low-density lipoprotein; HDL = high-density lipoprotein; Mets = metabolic syndrome; IFG = impaired fasting glucose; IGT = impaired glucose tolerance; T2D = type 2 diabetes; AUDIT = Alcohol Use Disorders Identification Test; LTPA = leisure-time physical activity; BDI = Beck's Depression Inventory; NSAIDs = non-steroidal anti-inflammatory drugs; IQR = inter-quartile range.

Table III shows the results of univariate and multivariate ordered logistic regression analyses relating baseline patient characteristics to the success in weight-loss outcome during the three-year follow-up. Newly diagnosed glucose disorder (odds ratio [OR] 1.37 [95% CI 1.02–1.84)] predicted success, whereas AUDIT-score ≥ 8 (OR 0.63 [95% CI 0.44–0.90]), BDI-score ≥ 10 (OR 0.61 [95% CI 0.42–0.90]) and number of drugs used (OR 0.91 [95% CI 0.83–0.99]) predicted poor outcome.

**Table III.** Ordered logistic regression analyses relating baseline subject characteristics to the success in weight-loss outcome.

	Univariate		Multivariate^a^	
Variables	OR (95% CI)	p-value	OR (95% CI)	p-value
Male sex	1.10 (0.83–1.46)	0.51		
Age	1.01 (0.99–1.03)	0.38		
Married or cohabiting	1.15 (0.80–1.64)	0.45		
Education years	1.00 (0.94–1.06)	0.88		
Hypertension	1.11 (0.83–1.48)	0.49		
Body mass index	1.02 (0.99–1.05)	0.27		
Total cholesterol ≥ 5.0 mmol/l	0.86 (0.65–1.15)	0.32		
Glucose disorder	1.34 (1.01–1.79)	0.048	1.37 (1.02–1.84)	0.038
Current smoker	0.80 (0.53–1.20)	0.28		
AUDIT-score ≥ 8	0.67 (0.48–0.95)	0.024	0.63 (0.44–0.90)	0.011
LTPA				
Low	1 (Reference)	0.93*		
Moderate	1.03 (0.73–1.46)			
High	0.98 (0.69–1.38)			
BDI score ≥ 10	0.62 (0.44–0.87)	0.006	0.61 (0.42–0.90)	0.012
Number of drugs used	0.89 (0.82–0.93)	0.004	0.91 (0.83–0.99)	0.034

Notes: ^a^Forward selection. Only those variables shown that entered the model. *P for linearity. AUDIT = Alcohol Use Disorders Identification Test; LTPA = leisure-time physical activity; BDI = Beck's Depression Inventory.

**Figure 1.**
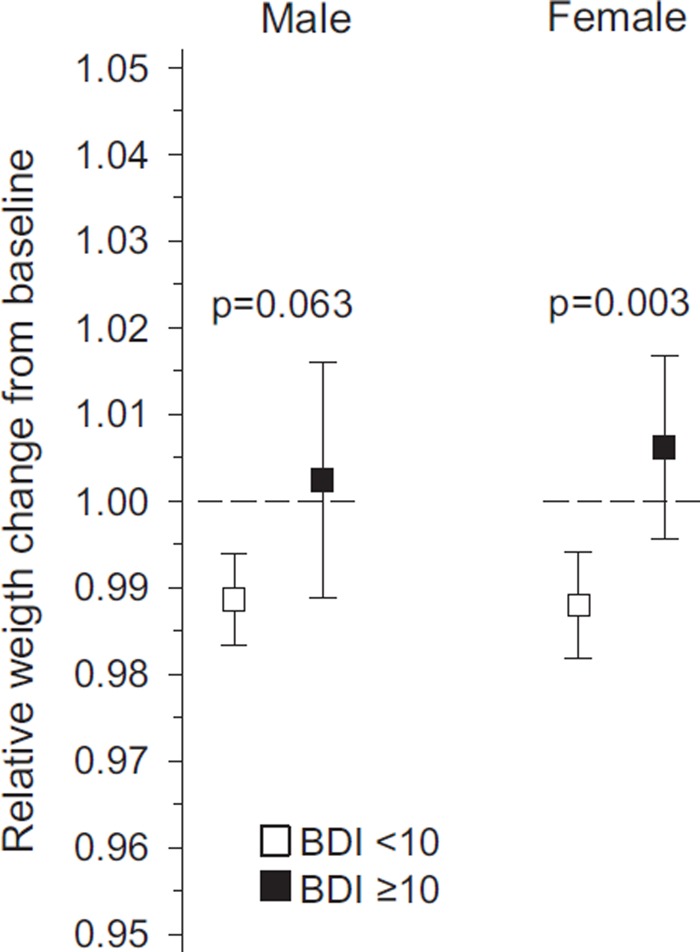
Relative weight change at year 3 in subjects with or without depressive symptoms. Note: BDI = Beck's Depression Inventory.

According to the BDI, of those who gained ≥ 5% weight during the follow-up, 33% had depressive symptoms at baseline. Age-adjusted relative weight change at year three in subjects with or without depressive symptoms is displayed in Figure 1.

## Discussion

This three-year follow-up study of primary care-based overweight and obesity screening programme showed that clinically meaningful weight loss can be achieved and maintained even with low-intensity counselling, which is possible to implement in a primary care setting. Some 18% of subjects lost at least 5% of their initial weight and managed to maintain the result for three years, which can be regarded as permanent change with numerous health benefits. In addition, 70% of the participants were able to stabilize their weight, which can be regarded as a clear profit at a time when the prevalence of sedentary lifestyle and obesity is increasing all over the world. Finkelstein et al. recently calculated that if adult obesity prevalence in the United States were to remain at 2010 levels, the savings in medical expenditures over the next two decades would be $549.5 billion ^[11]^.

With two treatment contacts in this screening-based programme, 18% of subjects lost 3–29 kg of their initial weight at three years. In behavioural treatment trials providing 12–26 intervention sessions during the first year, participants lost 4–7 kg at 12–18 months, compared with little to no weight loss in control groups [1]. These control groups were selected to represent usual care and could not receive a personalized intervention more frequently than annually [1]. The major limitation of our study is the lack of a control group. However, our intervention consisting of two brief individual lifestyle counselling sessions only at baseline compares with control groups in most primary care-relevant weight-loss intervention studies [1]. Because our study participants were also cardiovascular risk subjects, we considered it unethical not to provide all of them with self-care information. Another limitation of this study is that only 42% of the subjects attended the three-year follow-up visit. However, the differences between the followed-up and not followed-up subjects were not clinically meaningful and thus we think there was no selection bias in our study.

A meta-analysis of randomized trials comparing dietary counselling-based weight loss programmes with the usual care interventions showed that a mean net treatment effect was approximately 6% weight loss at year one, but half of the initial weight loss was typically regained after three years [12]. The fairly good long-term results of our study may be partly explained by the motivating impact of newly diagnosed glucose disorder. As a primary screening tool we used waist circumference, which is more informative for the patient than BMI, i.e. the ratio of weight and square of height. At the individual level, for any given BMI value, the subject with a larger waistline has more abdominal fat than the subject with a lower waist circumference [13]. Population studies have shown that people with large waist circumferences have an elevated health risk compared with those with normal waist circumferences within similar BMI categories [14–16].

Post et al. analysed the 2005–2008 US National Health and Nutrition Examination Survey data and showed that overweight and obese participants had an increased likelihood of having attempted to lose weight if they had been told by their physician that they were overweight [17]. However, only 45% of overweight and 66% of obese participants reported being told this by a physician [17]. Being overweight or obese is so common nowadays that a person is inclined to take the condition as “normal”. The expertise of nurses could be utilized more in primary care to identify and inform risk subjects in communities.

This study also revealed that one-third of the subjects who gained 5% or more weight during the follow-up had depressive symptoms at baseline. Among these subjects, excess alcohol use and regular drug usage were quite common. The evidence available on the baseline depression associated with weight loss maintenance is contradictory [18]. However, when recruiting individuals to weight-management programmes, screening for depression at enrolment would steer treatment efforts in an accurate direction before attempting to make changes in lifestyle.

In conclusion, by targeted screening it is possible to find overweight and obese subjects at increased cardiovascular risk in the community, and to induce clinically meaningful long-term weight loss or weight stabilization in a primary care setting. However, psychological factors are a critical component to consider before attempts are made to change the lifestyle of an overweight individual.
